# Plant Cryopreservation: A Look at the Present and the Future

**DOI:** 10.3390/plants10122744

**Published:** 2021-12-13

**Authors:** Carla Benelli

**Affiliations:** Institute of BioEconomy, National Research Council (CNR/IBE), Sesto Fiorentino, 50019 Florence, Italy; carla.benelli@ibe.cnr.it

Cryopreservation is known as an applied aspect of cryobiology or the study of life at low temperatures. Plant cryopreservation, specifically, is a process of cooling and storing vegetal structure as plant cells, tissues, or organs in liquid nitrogen (LN; −196 °C) or LN vapor (−160 °C). This methodology ensures the maintenance of samples’ viability after thawing, and indefinite storage is possible. The cryopreservation technique is based on the removal of all freezable water from tissues by physical or osmotic dehydration, followed by ultrarapid freezing. The ultralow temperature stops metabolic and biochemical reactions in the cell, after adequate dehydration of plant tissues, to prevent the formation of intracellular ice crystals, which can cause cell death and destruction of cell organelles during the freezing process. Cryopreservation is currently the most innovative and affordable biotechnological approach that allows safe long-term conservation of plant biodiversity without risk of genetic modifications. Few events and reports are available in the literature about genetic modifications in cryopreservation and, if they occurred, the exact mechanism and elucidation of the nature of genetic instability was not clarified, considering the multiple stages involved in the process (in vitro culture-cryoprotection-regeneration).

A pioneer in the plant cryopreservation was Prof. Akira Sakai, who reported the survival of mulberry twigs after exposure to liquid nitrogen [1]. However, plant cryopreservation studies took another 20 years to become established as an area of investigation. Since then, the cryopreservation has been disseminated by the development and application of cryogenic procedures, based on the slow cooling system first and then on the two-step cooling system. In particular, the two-step cooling system took over for its easy application, low cost, and less time-consuming procedure aiming at the direct immersion in liquid nitrogen of plant specimens from tissue culture, without resorting to expensive apparatus and with a considerable simplification of procedures. All this has allowed a large-scale application on plant species, with suitable cryopreservation protocols that can provide high plant regrowth after thawing, thus facilitating the establishment of organized and strategic cryobanks of plant genetic resources.

The two-step cooling process is based on the induction of explant “vitrification” during a very fast decrease in temperature [2].“Vitrification” of cells and tissues is the physical process, which avoids intracellular ice crystallization, during ultra-freezing, by the transition of the aqueous solution of the cytosol into an amorphous, glassy state. As a consequence of this process, plant tissues are protected from damage and remain viable during their long-term storage at −196 °C.

Various vitrification-based techniques have been developed and are available for different plant species such as vitrification, encapsulation-dehydration, encapsulation-vitrification, desiccation, [3] and, more recently, droplet vitrification and D or V cryo-plate [4,5], but the techniques are an ever-changing skill to improve the plant recovery rates, to expand the number of the cryopreserved species and, above all, working on the species, which are still hard to process with the cryopreservation. For example, in this Special Issue, present is an optimized cryopreservation protocol for embryonic axes of chestnut (*Castanea sativa* Mill.) developed based on the encapsulation–vitrification procedure; furthermore, the addition of activated charcoal (AC) as a component of the artificial matrix of synthetic seeds promoted growth by shortening the development times and limiting the loss of cryopreserved explants [6].

Cryogenic protocols are multi-stage, every step requires care to assure the successful of cryopreservation along with all the investigations connected to them, such as histo-anatomical, molecular, and physiological studies and in vitro culture procedures necessary to support recovery of cryopreserved explants.

The continuous research and technological evolution can markedly improve the cryogenic methodologies, allowing to enhance the recovery percentage of the species, as has occurred over the years. The new knowledge offers the prospect of bringing the cryopreservation technique to a superior level for preserving the vitality and integrity of the samples before and after storage. In *Stevia rebaudiana*, for example, the effectiveness of shoot tips cryopreservation increased with the application of V cryo-plate procedure, resulting in superior regrowth of 93%, [7] compared to the vitrification procedure applied by Shatnawi et al. [8], which obtained 68% of shoot tips regrowth. Moreover, new information has been acquired based on investigations in order to improve the explant physiological state, pre-treatment conditions, time and conditions of the cryoprotectant treatments, enhance the cooling and warming rates, and the recovery medium to achieve successful viability and regrowth of cryopreserved species. A contribution to the arduous process of optimizing cryoprotectant formulations in this Special Issue was given. Faltus at al. [9] has described in detail the thermal characteristics of two important plant vitrification solutions (PVS2 and PVS3) and their components depending on their concentration and temperature, while the opportunity of PVS2 modification to provide better application to new species has been dealt with by Zamecnik et al. [10]; support can also come from the Coherent Anti-Stokes Raman Scattering (CARS) microscopy by facilitating the visualization of deuterated cryoprotectants within living cells [11].

Distinct and adapted cryogenic techniques have been applied to a wide range of explants, including pollen, seeds, somatic and zygotic embryos, suspension or callus culture, apical buds, shoot tips, and dormant buds. The explant choice is also connected to the best in vitro recovery method for a specific genotype after cryopreservation. Hence, it is necessary to have an efficient in vitro regeneration system for a wider application of plant cryopreservation. For vegetatively propagated species, the most widely used organs are shoot tips excised from the in vitro plant [12,13]. Using this type of explant, the somaclonal variation is less probable to occur with respect to direct or indirect organogenesis [14]. In some woody species (e.g., apple, pear), using dormant buds as explants has also been developed and applied to cryopreservation protocols, assessing the recovery by grafting [15,16]. The dormant bud cryopreservation technique is an efficient alternative to the labor-intensive in vitro shoot tip cryopreservation process, allowing the preservation of large quantities of germplasm in a season [17]. Recently, in the 23 cryopreserved blackcurrant cultivars, using non-desiccated dormant buds collected from a greenhouse, the estimated recovery ranged between 42 and 90% [18].

Over time, cryopreservation protocols have been established for several hundreds of plant species [3] and further research is being conducted to enable adoption of this approach even more broadly. Currently, over 10,000 accessions starting from in vitro cultures are preserved through cryopreservation methods, and more than 80% of these belong to five crops: potato, cassava, bananas, mulberry, and garlic. Other important plant cryopreservation collections representing thousands of accessions are those of dormant apple buds [19,20,21]. Cryopreservation techniques are now used for plant germplasm storage in many institutes around the world [22,23]. The preservation of plant genetic resources (PGRs) is highly important for food security and agrobiodiversity, in breeding programs to obtain new or more productive plants, but also to have plants resistant to abiotic and biotic stresses. The application of advanced biotechnology, such as cryopreservation, represents an efficient alternative method for ex situ conservation of germplasm, and helps overcome several limitations of storage by conventional methods (seed banks and clonal orchards) [24,25,26]. Cryopreservation can be considered a safe strategy for long-term conservation and a backup to field collections to reduce the loss of plant germplasm. In this Special Issue, a protocol for seed cryopreservation and following in vitro germination has been reported for the first time in Eastern Turkeybeard (*Xerophyllum asphodeloides* (L.) Nutt.), a threatened species that has responded positively to the cryogenic technique [27].

On the other hand, a clear indication that the cryopreservation is a useful and necessary tool for conservation of plant species has also been underlined in the Plant Conservation Report 2020 [28]; this report mentioned the cryopreservation among alternative conservation methods. Several cryopreservation germplasm repositories (cryobanks) have been established for various plant species in different countries (e.g., cassava, potato, banana, apple, pear, coffee, mulberry, garlic), applying different cryopreservation techniques, and this strategy represents a guide for conservation in the future.

However, a few remarks should be made to face this new challenge; specifically, some critical issues need to be overcome and they will be part of the strategy to be pursued over the coming years. Detailed and exhaustive reviews [3,12,29,30,31] and articles described the various cryogenic methods applied to plants, but although much progress has been accomplished in the last years, some drawbacks still limit the wide use of cryopreservation, and the difficulties and challenges with the aim to further expand its frontiers should be considered.

The cryopreservation practice requires an initial technological investment but the maintenance costs for the application of the different techniques will later be lower, considering that the use of cryopreservation facilitates the storage and rapid multiplication of plant germplasm in a pathogen-free aseptic environment as well as optimization of physical space and labor. In a perspective of conservation strategy, the introduction of an accession into cryopreserved storage is more expensive than establishing an accession in in vitro culture or in the field, but the cryopreservation costs for the long-term vision (over 20 years) are considerably lower than those of maintenance in the field or in vitro, particularly when many accessions are preserved.

The difficulty to transfer technology and validating protocols between laboratories is a key issue [32]. There are some critical factors that involve all the cryopreservation steps such as type of plant materials, conditions of preculture, cryopreservation technique, cooling, warming, and regrowth conditions [13]. The lack of reproducibility available protocols, or the difficulty in adapting them, can be due to numerous causes, ranging from different sources of laboratory supplies to the different equipment and the different levels of technical skills found in cryopreservation laboratories.

The development and dissemination of increasingly simple and well-described protocols [33], with adequate facilities and trained personnel, will allow new challenges in each cryopreservation laboratory or institution as well as the implementation of the cryopreservation procedures in cryobanks and for biological materials and organisms important for research and in applications, including algae [34].

For this reason, it will therefore be essential to carry out joint strategies and programs among different countries to overcome some critical issues and, above all, find large-scale, simple and effective protocols and easily replicable in all laboratories. Accumulating experience in routine procedures, enhancing the skills of staff can be helpful, in addition to proceed with basic research, which should be encouraged (or rather financed) to achieve a better understanding of some topics less investigated.

In conclusion, since there is an increasing importance of plant preservation technology in the modern world, a clear need exists to have reliable methodologies as well as strong research and development activity in cryopreservation to ensure the applications are fit for purpose. The development of simple, reliable and cost-effective methods is essential and advances will be made faster if the know-how will be wide and shared, all this will support the cryopreservation, which is potentially the safest method to maintain vegetative germplasm and recalcitrant seed for a long-time life.
